# Elastocaloric effect and superelastic stability in Ni–Mn–In–Co polycrystalline Heusler alloys: hysteresis and strain-rate effects

**DOI:** 10.1038/s41598-017-02300-3

**Published:** 2017-05-18

**Authors:** Binfeng Lu, Jian Liu

**Affiliations:** 1 School of Chemistry and Materials Engineering, Jiangsu Key Laboratory of Advanced Functional Materials, Changshu Institute of Technology, Changshu, Jiangsu 215500 China; 2 Key Laboratory of Magnetic Materials and Devices, Ningbo Institute of Material Technology and Engineering, CAS, Ningbo 315201 China; 3 Zhejiang Province Key Laboratory of Magnetic Materials and Application Technology, Ningbo Institute of Material Technology and Engineering, CAS, Ningbo 315201 China

## Abstract

Controlling material hysteresis and working frequency variability are fundamentally important for refrigeration cycle efficiency and power density in solid-state cooling systems. For elastocaloric cooling, understanding the relationship between the width of the stress hysteresis and elastocaloric behaviour during superelastic cycles under varied strain rates is important. In this work, we report the effects of strain rate effects on the superelastic and elastocaloric responses in Ni_45_Mn_50−x_In_x_Co_5_ (x = 13.6–14.0 in at%) polycrystalline alloys. We observed a strong correlation between stress hysteresis and superelastic stability during mechanical cycling under increasing strain rates. Excellent transformation reversibility and stable superelastic responses are observed for x = 13.6 with a narrow hysteresis (49 MPa), whereas transformation irreversibility and dramatically deteriorated superelastic stability occur for x = 13.8, with a wide hysteresis (138 MPa). Furthermore, isothermal loading–unloading cycles under increasing and constant maximum applied stress were performed for the x = 13.6 samples, with a combination of low transformation stress and small transformation hysteresis. We suggest that a balance between transformation strain and hysteresis energy loss is fundamental to achieving a high coefficient of performance for elastocaloric materials.

## Introduction

Because of priorities in energy savings and eco-friendliness, novel solid-state refrigeration techniques based on the caloric effect from solid-state transformation driven by external stimuli have been proposed, as potential alternatives to conventional vapour compression cooling and are attracting significant attention^1–5^. Recently, the elastocaloric effect (eCE), primarily stemming from lattice vibrations or softening entropy changes, has been reported in conventional shape-memory alloys (SMAs) (e.g., Cu–Zn–Al^6, 7^, Cu–Al–Mn^8^, Ni–Ti–(Cu,Co)^9–15^, Fe–Pd^16, 17^) and Heusler-type magnetic or metamagnetic SMAs (including Ni–Fe–Ga–(Co)^18–20^, Co–Ni–Ga^21^, Ni–Mn–In–(Co)^22–24^, Ni–Mn–Sn–(Co)^25, 26^ and Ni–Mn–Sb–Co^27^). In terms of an elastocaloric cooling system, progress has been made in developing an elastocaloric prototype cooler (using Ni–Ti SMA ribbon as refrigerant)^13^, optimizing the cooling system design via numerical simulation^28^, and achieving a system coefficient of performance (COP, as high as 7) superior to that for magnetocaloric Gd^15, 29^.

From the perspective of practical cooling applications, the combination of a large, reversible caloric effect and excellent mechanical stability under field cycling is critical to achieve a high cooling efficiency for long-term service. To achieve a large caloric effect, materials with a first-order phase transition (FOPT) are desirable because,a FOPT accompanies latent heat. However, the effects of a FOPT are twofold: the giant caloric effect usually associated with a large hysteresis. Hysteresis, as a direct measure of the dissipated work, results in energy loss, a reduction of the caloric effect during cycling^14^ and a decrease in the efficiency of cooling systems^30, 31^. Given that hysteresis in SMAs originates from elastic incompatibilities between the martensite and austenite phases^32^, the alloy composition can be tuned to meet the strong geometry compatibility requirements, leading to narrow thermal hysteresis and excellent transformation reversibility^33–35^. On the other hand, operational frequency, manifested by the rate at which the driving field is applied or removed, is an important parameter in solid-state cooling systems because it correlates the system heat exchange, temperature relaxation^36, 37^ and cooling power (COP, and exergy efficiency)^38^. Analogous to electric/magnetic field frequency in an electrocaloric/magnetocaloric refrigeration cycle, the stress frequency in an elastocaloric cooling cycle can be reflected by the strain rate during loading and unloading.

In terms of Heusler-type magnetic SMAs, most studies have focused on the compositional sensitivity of the thermal hysteresis in Ni–Mn–In and, Ni–Mn–Sn–Co polycrystalline alloys^39, 40^, and on the temperature dependence of the stress hysteresis in single crystalline Ni–Mn–In–Co^41, 42^. Admittedly, the compositional effect on the stress hysteresis in polycrystalline magnetic SMAs remains unclear. Meanwhile, variations in the strain rate are desirable to achieve elastocaloric cooling with variable frequency, and high power density is expected with a high frequency. However, the effect of stress hysteresis on superelasticity cycling stability under various strain rates has not been clarified.

In this paper, we investigate the composition and strain-rate effects on the superelastic response and elastocaloric effect in Ni–Mn–In–Co polycrystalline alloys. We observed a significant correlation between the stress hysteresis and superelastic stability under mechanical cycling with increasing strain rates. We found that a narrower stress hysteresis resulted in greater superelastic stability. Furthermore, isothermal loading–unloading cycling under increasing and constant maximum applied stress was conducted for Ni_45_Mn_50−x_In_x_Co_5_ (x = 13.6). The x = 13.6 samples exhibt a combination of relatively low transformation stress and small transformation hysteresis. We infer that a balance between transformation strain and hysteresis energy loss is fundamental to achieving a high COP for elastocaloric materials.

## Results and Discussion

### Thermally driven and isothermal stress-induced martensite transformation

**Figure 1:**
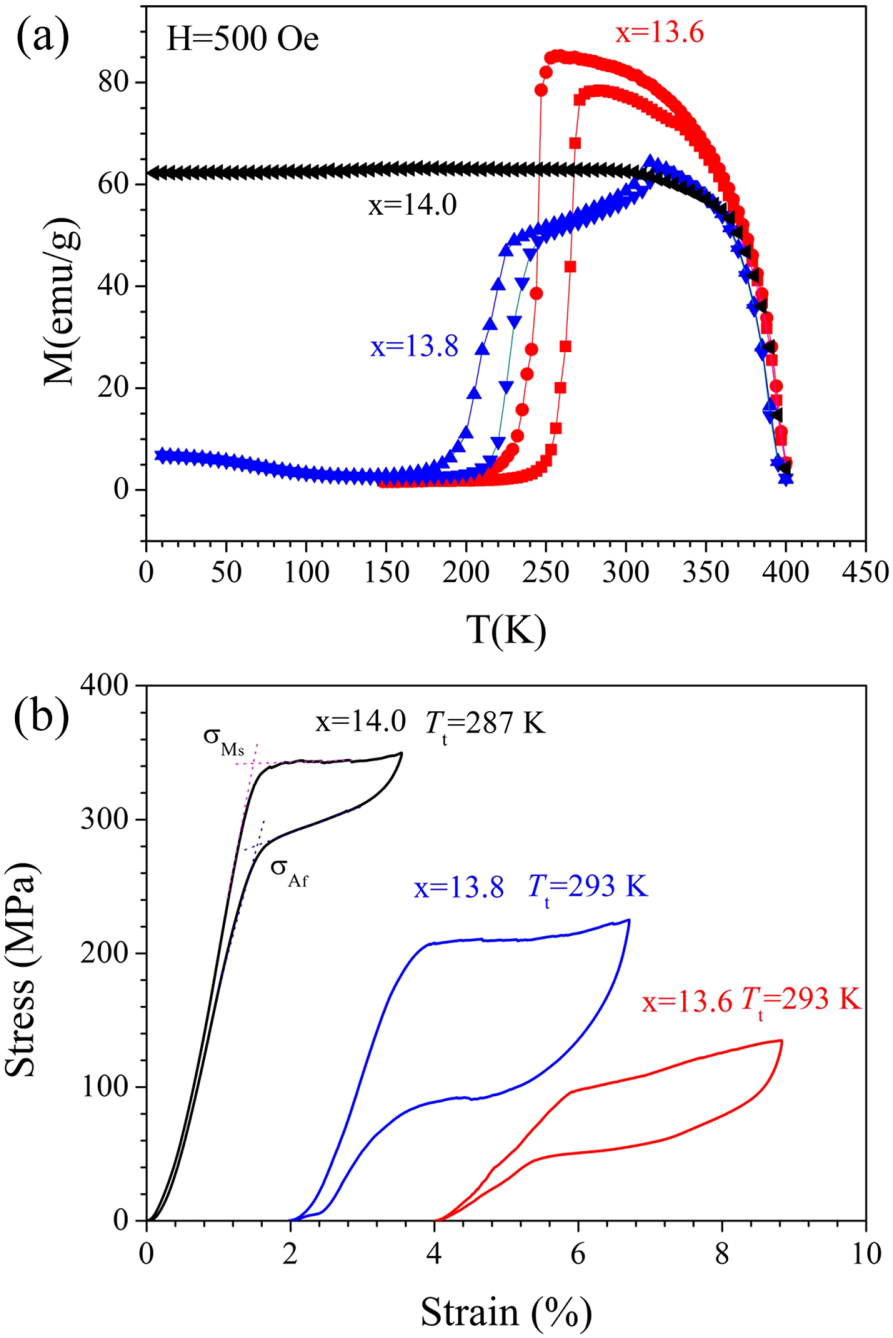
(**a**) Temperature dependence of the magnetization in a magnetic field of 500 Oe for polycrystalline Ni_45_Mn_50−x_In_x_Co_5_ (x = 13.6, 13.8, 14.0) alloys. (**b**) Isothermal stress–strain response upon loading and unloading of Ni_45_Mn_50−x_In_x_Co_5_ (x = 13.6, 13.8, 14.0) samples at a strain rate of 0.0003 s^−1^. The horizontal ordinate is offset by 2% and 4% for x = 13.8 and x = 14.0, respectively.

Figure 1(a) shows the magnetization versus temperature (*M*–*T*) curves for Ni_45_Mn_50−x_In_x_Co_5_ (x = 13.6, 13.8, 14.0 in at%) alloys under a magnetic field of 500 Oe. For the x = 13.6 sample, upon heating, the nonmagnetic martensite transforms to ferromagnetic austenite in a temperature range between the austenite start temperature *A*_s_ = 261.4 K and the austenite finish temperature *A*_f_ = 268.4 K. Upon cooling, martensite starts to form at *M*_s_ = 247.8 K and finishes at *M*_f_ = 241.4 K. The resultant temperature span for the forward or reverse martensite transformation (MT) upon heating or cooling is ~7 K. With an increase in the indium content (x = 13.8), the characteristic transition temperatures shift toward a lower temperature, whereas a much wider temperature span (~28 K and 26 K) exists for the forward and reverse MTs, respectively. By contrast, further increases to the indium content (x = 14.0) causes the ferromagnetic austenite to be retained even when the sample is cooled to 5 K, suggesting that no thermally induced forward martensitic transformation occurs during cooling. Such a phenomenon illustrates that the MT can be completely blocked, even upon cooling under a weak magnetic field of 500 Oe, as predicted by the correlation between the transformation entropy change Δ*S*_tr_ and the difference between the Curie and MT temperatures (*T*_c_ − *M*_s_)^43^. The corresponding (*T*_c_ − *M*_s_) values for x = 13.6 and x = 13.8 reach 147 K and 172 K, respectively (as listed in Table 1) and the expected (*T*_c_ − *M*_s_) for x = 14.0 should be approximately 200 K according to the linear correlation between *M*_s_ and the valence electron concentration (as a function of indium content x)^44^. Correspondingly, the Δ*S*_tr_ for x = 14.0 with a (*T*_c_ − *M*_s_) of ~200 K could be rather small because of the counterbalancing effect between the magnetic and lattice contributions for the transformation entropy change in Ni–Mn–In–Co metamagnetic SMAs^45^.

**Table 1:** Phase transition temperatures (*M*_s_, *M*_f_, *A*_s_, *A*_f_) from ther *M*–*T* measurements, the interval between the Curie and MT temperatures (*T*_c_ − *M*_s_), and the testing temperature *T*_t_ for the isothermal stress–strain behaviours in Ni_45_Mn_50−x_In_x_Co_5_ samples (x = 13.6, 13.8, 14.0).

Composition (at. %)	*M*_s_ (K)	*M*_f_ (K)	*A*_s_ (K)	*A*_f_ (K)	*T*_c_ − *M*_s_ (K)	*T*_t_ (K)
x = 13.6	247.8	241.4	261.4	268.4	147	293
x = 13.8	222.9	195.4	217.4	235.5	172	293
x = 14.0	—	—	—	—	—	287

Unlike that in thermally induced MT behaviour, all three compositions exhibit stress-driven superelasticity near room temperature (as presented in Fig. 1(b)). The testing temperature (*T*_t_) for x = 13.6 and x = 13.8 is 293 K. For x = 14.0, a lower *T*_t_ (287 K) is used to decrease the critical transformation stress (CTS) and to avoid yielding or cracking of the austenite phase. As demonstrated in the stress–strain curve corresponding to x = 14.0, the critical stresses for the start of the forward transformation (*σ*_Ms_) and for the completion of the reverse transformation (*σ*_Af_) are determined from the crossing points of the lines linearly extrapolated from the loading/unloading elastic curve and the forward/reverse transformation plateaus, respectively. The corresponding stress hysteresis (*σ*_hys_) is defined as the difference between *σ*_Af_ and *σ*_Ms_, whereas the hysteresis energy loss per unit volume (*E*_loss_) during loading–unloading cycling is the area enclosed by the hysteresis loop in the stress-strain curve.

As shown in Table 2, the CTS for martensite transformation *σ*_Ms_ varies from 96 MPa (x = 13.6) to 208 MPa (x = 13.8) to 342 MPa (x = 14.0), whereas the stress hysteresis *σ*_hys_ evolves from 49 MPa (x = 13.6) to 138 MPa (x = 13.8) to 63 MPa (x = 14.0). That is, to achieve a comparable transformation strain (~3%), the driving stress is low and the stress hysteresis is small for x = 13.6, whereas the driving stress is higher and the stress hysteresis is much larger for x = 13.8. For x = 14.0, the driving stress is the highest, whereas the stress hysteresis is moderate, with a transformation stain of 2%.

**Table 2:** The critical transformation stresses *σ*_Ms_ and *σ*_Af_, stress hysteresis *σ*_hys_ = (*σ*_Ms_ − *σ*_Af_), transformation strain ε_tr_, total work done on the specimen *E*_1_, energy density per unit volume which is stored and available upon unloading *E*_2_ and the hysteresis energy loss *E*_loss_ = (*E*_1_ − *E*_2_) in Ni_45_Mn_50−x_In_x_Co_5_ (x = 13.6, 13.8, 14.0) alloys.

Composition (at. %)	*σ*_Ms_ (MPa)	*σ*_Af_ (MPa)	*σ*_hys_ (MPa)	*ε*_tr_ (%)	*E*_1_ (MJ/m^3^)	*E*_2_ (MJ/m^3^)	*E*_loss_ (MJ/m^3^)
x = 13.6	96	47	49	3.0	4.3	2.6	1.7
x = 13.8	208	70	138	3.1	7.9	4.2	3.7
x = 14.0	342	279	63	2.0	9.1	7.9	1.2

The MT phenomenon in the case of x = 14.0 occurs via the application of an external uniaxial stress while not being cooling at zero stress and can be understood from the energy landscape for the austenite and martensite phases^46^. The free energies of the two phases are functions of temperature and stress and generally involve several sources (chemical free energy Δ*G* ^ch^, mechanical energy *E*_m_, interfacial energy *E*_i_, surface energy *E*_sf_, stored elastic strain energy *E*_el_ and frictional work *E*_fr_). The corresponding expression for the change in the Gibbs free energy (per unit volume) of a sample during transformation is shown as^46^:

1 $${\rm{\Delta }}{G}_{A\leftrightarrow M}=[{\rm{\Delta }}{G}^{ch}-\sigma \varepsilon ]\delta +[{\gamma }_{i}{A}_{i}+{\rm{\Delta }}{\gamma }_{sf}{A}_{sf}+{E}_{el}]\delta +{E}_{fr}$$

where *γ*_*i*_ is the austenite/martensite interface energy per unit area, *A*_*i*_ is the interfacial area, Δ*γ*_sf_ is the difference in the surface energy per unit area, *A*_sf_ is the specific surface area and  *E*_el_ is the average increment in elastic strain energy density. The frictional work *E*_fr_ is irreversible and is positive in both transformation directions. For equation (1), the fraction of martensite *δ*∈(0, 1) is positive during forward transformation whereas *δ*∈(0, −1) is negative during reverse transformation. For x = 14.0, the application of a uniaxial compressive stress leads to a decreasing shift in the Gibbs free energy curve for martensite *G*_M_(*T*), which in turn results in a higher equivalent temperature *T*_0_. Here, *T*_0_ denotes the equilibrium temperature at which ${\rm{\Delta }}{G}_{{\rm{A}}}^{ch}={\rm{\Delta }}{G}_{{\rm{M}}}^{ch}$. When the input work (the applied compressive stress multiplied by the strain) is sufficiently large to overcome the energy barrier for forward martensitic transformation, stress-induced martensitic transformation can occur even at room temperature, which is far above the stress-free equivalent temperature (as presented in Fig. 1(b)).

### Temperature–time profiles: strain-rate effect

To investigate the composition and strain-rate effects on the elastocaloric effect, direct measurements of the temperature change are conducted for Ni_45_Mn_50−x_In_x_Co_5_ (x = 13.6, 13.8, 14.0) alloys at a series of strain rate ranging from 0.003 to 0.03 s^−1^ (1–10 mm min^−1^).

**Figure 2:**
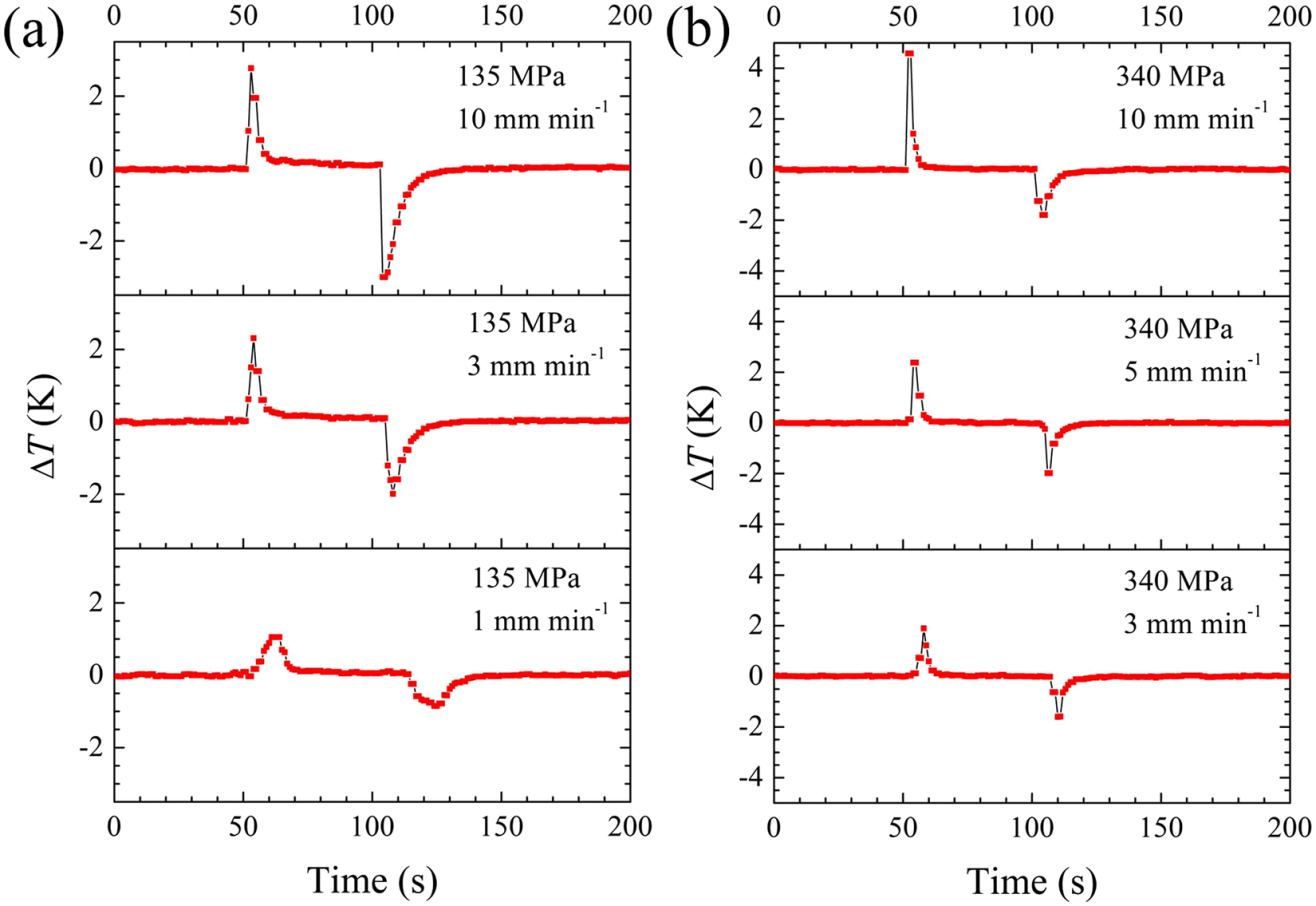
Strain-rate dependence of temperature-time profiles for narrow- stress-hysteresis Ni_45_Mn_50−x_In_x_Co_5_ samples at room temperature: (**a**) x = 13.6, *T*_test_ = 293 K, and (**b**) x = 14.0, *T*_test_ = 287 K.

Initially, we focused on the strain-rate sensitivity of the elastocaloric effect for compositions with relatively narrow stress hysteresis (x = 13.6 and x = 14.0). Figure 2 shows the temperature change as a function of time at typical strain rates for Ni_45_Mn_50−x_In_x_Co_5_ (x = 13.6, 14.0) samples at approximately room temperature. To avoid possible cracking in this brittle material, the maximum stress was only set at 135 MPa and 340 MPa for x = 13.6 and x = 14.0, respectively. In the case of x = 13.6, the temperature changes upon both loading (Δ*T* ^loading^) and on unloading (Δ*T* ^unloading^) are clearly strain-rate dependent. Using unloading as an example, we observe that the magnitude of Δ*T* ^unloading^ increases from 0.8 to 2.0 to 3.0 K as the loading condition varies from 1 to 3 to 10 mm min^−1^ (Fig. 2(a)). By contrast, the elastocaloric effect in x = 14.0 generally exhibits less sensitivity to the strain rate, except for a large value of Δ*T* ^loading^ (+4.6 K) at a loading rate of 10 mm min^−1^. As the applied condition changes from 3 to 5 to 10 mm min^−1^, the magnitude of Δ*T* ^unloading^ evolves from 1.6 to 2.0 to 1.8 K (as shown in Fig. 2(b)).

**Figure 3:**
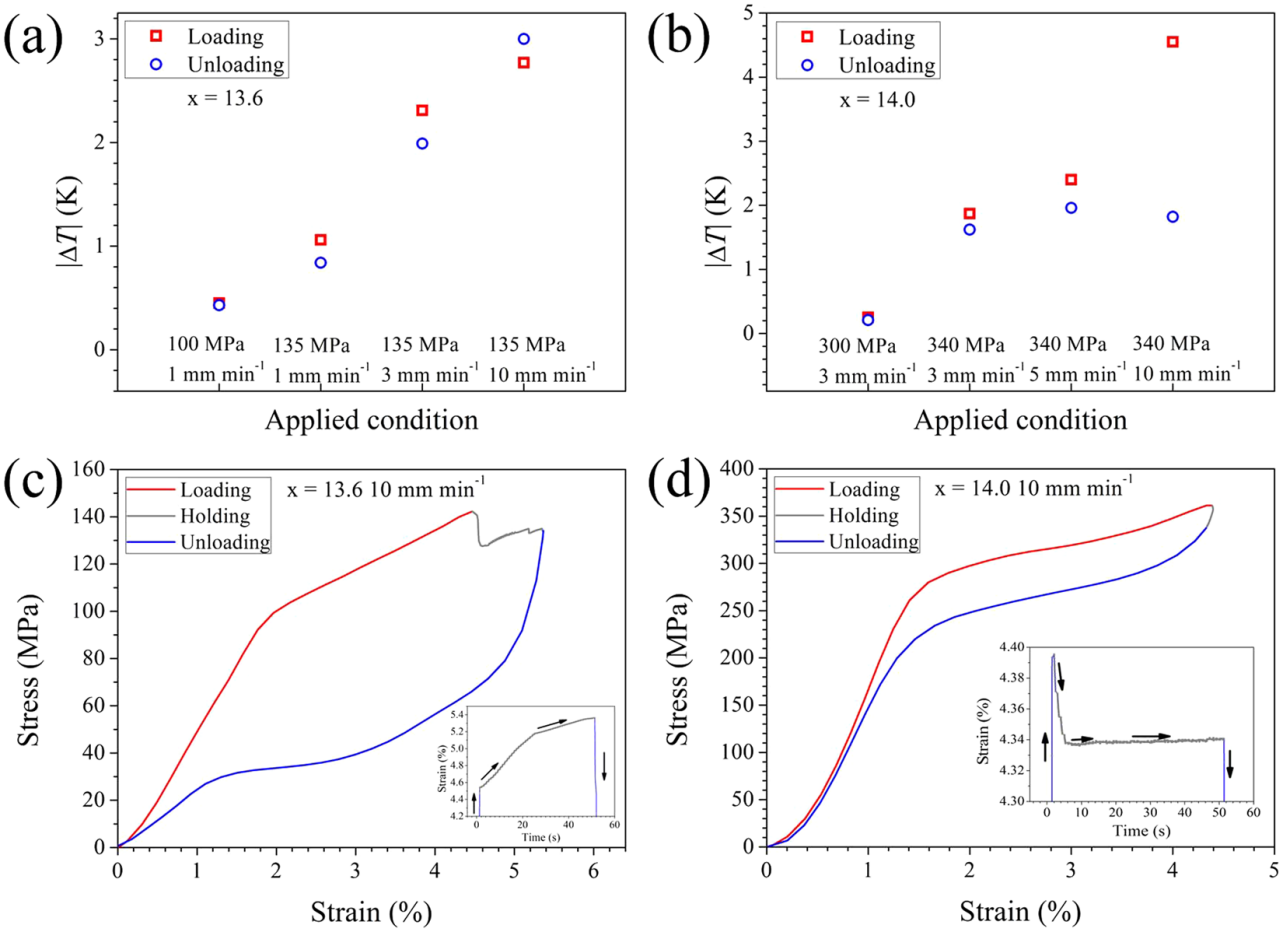
Strain-rate dependence of the maximum temperature changes upon loading and unloading ((**a**) x = 13.6, *T*_*test*_ = 293 K and (**b**) x = 14.0 *T*_test_ = 287 K) and corresponding quasi-adiabatic stress–strain responses at 10 mm min^−1^ ((**c**) x = 13.6 and (**d**) x = 14.0) for Ni_45_Mn_50−x_In_x_Co_5_ samples at room temperature. The insets in 3(**c**) and 3(**d**) show corresponding strain–time responses during the loading, holding and unloading processes.

The temperature changes upon loading and unloading, Δ*T* ^loading^ and Δ*T* ^unloading^, under different strain rates are systematically plotted in Fig. 3(a) and (b), respectively. One thing in common for x = 13.6 and x = 14.0 is that Δ*T* ^loading^ is always slightly larger than Δ*T* ^unloading^ at moderate strain rates (no more than 5 mm min^−1^). This phenomenon is attributed to frictional heating (the release of friction heat generated from martensite/austenite and martensitic variants’ interfaces), which enhances the heating effect upon loading but reduces the cooling effect upon unloading. At a higher strain rate (10 mm min^−1^), the magnitude of Δ*T* ^unloading^ (3.0 K) exceeds that of Δ*T* ^loading^ (2.8 K) for x = 13.6, whereas │Δ*T* ^loading^│ ( = 4.6 K) is substantially larger than │Δ*T* ^unloading^│( = 1.8 K) for x = 14.0.

To clarify the possible sources of the abnormal elastocaloric temperature change behaviour, corresponding stress–strain and strain–time responses under quasi–adiabatic condition (10 mm min^−1^) during loading, holding and unloading processes are presented in Fig. 3(c) and (d). For x = 13.6, on loading, stress-induced transformation (SIM) begins at ~1.9% and ceases at ~4.5%, resulting in a transformation strain of 2.6%. During the isothermal holding process, SIM continues as the total strain increases to 5.3%, leading to a net transformation strain of 0.8%. Upon loading, a higher transformation strain (3.4%) is expected because the superelastic loop is complete. The elastocaloric effect in SMAs with a first-order phase transition originates from released or absorbed heat during forward and reverse martensitic transformation. Therefore, the magnitude of the adiabatic temperature change Δ*T* should be related to fraction of the reversible transformation phase d*f* during mechanical cycling. In terms of the correlation between Δ*T* and d*f*, the theoretical formula shows the concept of fractional temperature change (Δ*T* = *τ* d*f*, where *τ* is the maximum temperature change assuming the sample experienced a complete transition, and d*f* is the fraction of transformation phase)^47^. Meanwhile, the magnitude of the reversible transformation phase d*f* is expected to affect the macroscopically transformation strain *ε*_tr_. Likely, the cooling effect from additional transformation strain on unloading exceeds the heating effect from frictional heating. That is, the contribution from the increased transformation strain (temperature drop) may play a more significant role than that from friction heat (temperature rise), leading to a relatively larger Δ*T* for x = 13.6 upon unloading at 10 mm min^−1^. *In-situ* X-ray measurements are necessary in future work to determine the transformation strain, because lattice deformation under uniaxial stress can be determined from lattice parameters as obtained via *in situ* high-energy X-ray diffraction experiments^48^.

In the case of x = 14.0, the asymmetric temperature–time profile cannot be due to transformation irreversibility because the strain is fully recovered upon unloading. Meanwhile, the variation of transformation strain during holding is negligible (~0.06%), suggesting that the latent heat released on loading is comparable to that absorbed upon unloading. Interestingly, detailed information about the strain–time response (as plotted in the inset of Fig. 3(d)) shows that strain overshooting occurs during the loading portion, followed by a slightly smaller but constant strain, quite different from the creep-like behaviour observed in the case of x = 13.6 (inset of Fig. 3(c)). This loading-induced strain overshooting corresponds to a stress variation from 0 to 362 MPa within 1.5 s, implying a nearly adiabatic deformation condition. Notably, both the adiabatic deformation and latent heat of martensitic transformation contribute to the elastocaloric temperature change, as verified in Ni–Ti SMA^49^. Therefore, the heating effect from adiabatic deformation manifested by strain overshooting may be the responsible for the abnormal large Δ*T* ^loading^ in x = 14.0 at a high strain rate.

**Figure 4:**
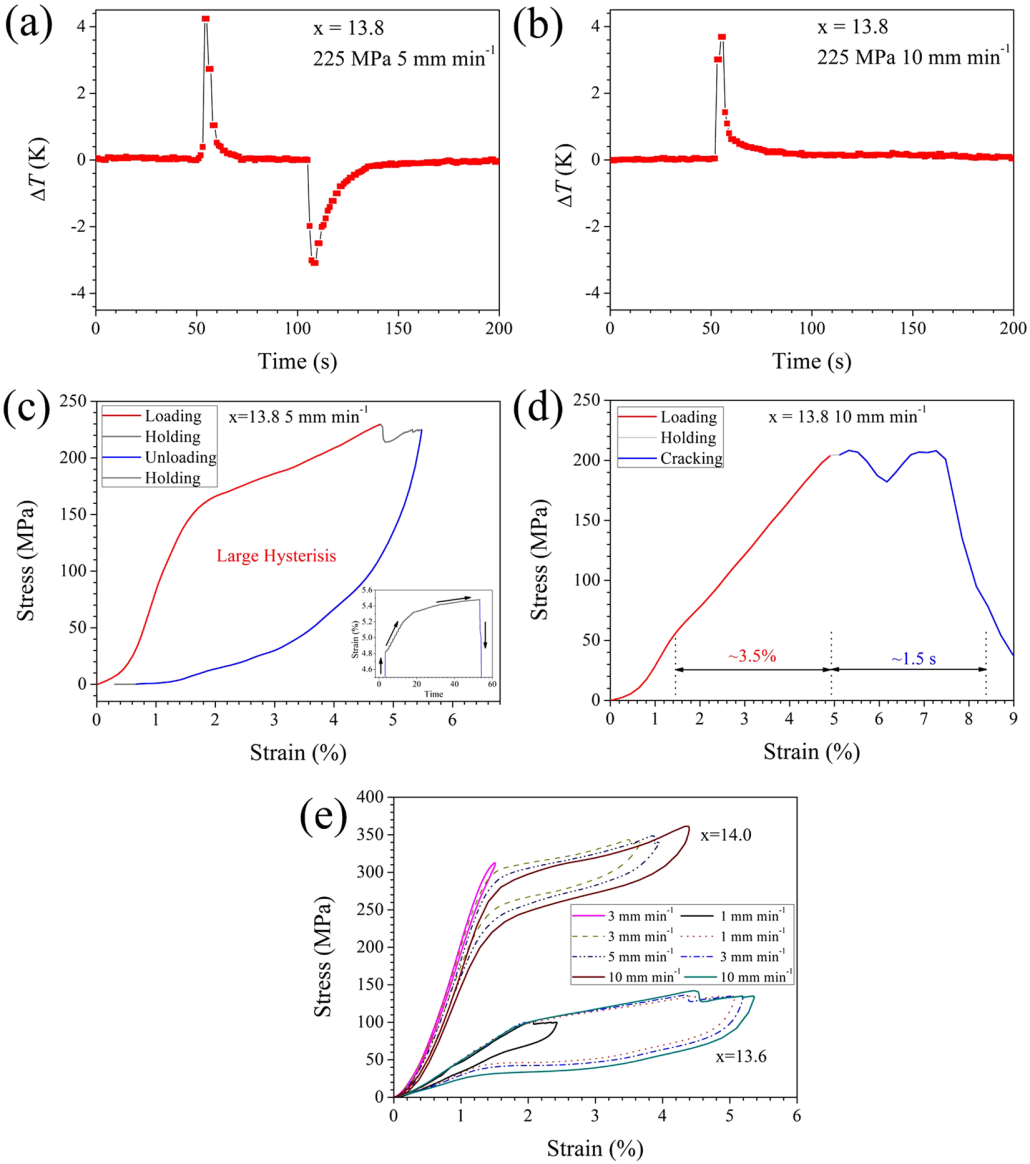
Typical quasi-adiabatic temperature–time profiles and stress–strain responses for large-stress-hysteresis Ni_45_Mn_50−x_In_x_Co_5_ samples (x = 13.8): (**a**,**c**) 5 mm min^−1^ and (**b**,**d**) 10 mm min^−1^. The insets in (**c**) show the corresponding strain–time responses during the loading, holding and unloading processes. The evolution of the stress-strain responses for the x = 13.6 and x = 14.0 samples at room temperature is shown in (**e**), wherer the applied conditions correspond to those in Fig. 3(a) and (b), respectively.

Here, we focus on another Ni–Mn–In–Co alloy (x = 13.8), that exhibit stress hysteresis, even larger than the sum of stress hysteresis in x = 13.6 and x = 14.0 (shown in Fig. 1(b)). As plotted in Fig. 4(a), fast loading to 225 MPa at a loading rate of 5 mm min^−1^ results in a temperature rise of 4.2 K, which is much larger than that (2.8 K) for x = 13.6 upon loading even at 10 mm min^−1^. Upon unloading, a temperature drop of 3.1 K is observed. To investigate the origin in the asymmetry of the temperature–time profile, we analysed the details of the corresponding stress–strain and strain–time responses (Fig. 4(c)). The transformation strain upon loading is 3.3%. The increased transformation strain (~0.7%) due to strain relaxation from 4.8% to 5.5% during holding is exactly counterbalanced by the unrecovered transformation strain upon unloading (0.7%). As a result, the transformation strain upon loading is comparable to that upon unloading. Because transformation strain reflects the latent heat, the expected latent heat released upon loading and that absorbed upon unloading should be equivalent. Meanwhile, no strain overshooting is observed upon loading, suggesting no contribution from adiabatic deformation to Δ*T* ^loading^. Thus, we believe that frictional heating should play a significant role in increasing the Δ*T* ^loading^, decreasing the Δ*T* ^unloading^, and finally a large difference between them (1.1 K).

At a higher strain rate (10 mm min^−1^), a temperature rise of only 3.7 K can be achieved upon loading, because of sudden cracking of the sample at the beginning of holding (Fig. 4(b) and (d)). Comparing the stress–strain responses in Fig. 4(c) and (d) reveals that cycling at increasing strain rates (from 5 to 10 mm min^−1^) leads to superelastic behaviour instability. The critical transformation stress decreases dramatically from 156 MPa to 51 MPa, whereas the transformation stress slope steepens from 22.4 MPa %^−1^ to 45.5 MPa %^−1^. In addition, the hysteresis energy loss at 5 mm min^−1^ reaches approximately 6.2 MJ m^−3^.

### Cycling stress–strain responses with increasing strain rates

From the perspective of elastocaloric cooling applications, the cycling stability of the elastocaloric effect manifested by the superelasticity behaviour at varying strain rates is equally important as the magnitude and reversibility of elastocaloric effect.

To systematically compare the evolution of the cycling superelastic stability, we examine the stress–strain responses with one-to-one correspondence to temperature–time responses for different strain rates, as shown in Fig. 2. Figure 4(e) summarizes the evolution of stress-strain responses with increasing strain rate for Ni_45_Mn_50−x_In_x_Co_5_ (x = 13.6, 14.0) samples at room temperature. Unlike x = 13.8, both compositions with narrow stress hysteresis generally show relatively stable superelastic behaviour. In the case of x = 14.0, the critical transformation stress evolves from ~301 MPa (at 1 mm min^−1^) to 288 MPa (at 10 mm min^−1^), whereas the transformation stress slope varies from ~17 MPa %^−1^ (at 1 mm min^−1^) to 25 MPa %^−1^ (at 10 mm min^−1^). Interestingly, x = 13.6 exhibits excellent superelastic stability as both the transformation stress and transformation plateaus at 1 mm min^−1^ nearly reproduce that at 3 mm min^−1^. Such obvious differences in cycling superelastic stability between x = 13.6 and x = 13.8 (in Fig. 4), are likely related to different geometry compatibility. Because geometry compatibility can be reflected by the magnitude of the (isothermal) hysteresis, x = 13.6 with a narrow stress hysteresis is reasonably inferred to possess better geometry compatibility than x = 13.8 with wide stress hysteresis. Such a high composition sensitivity of superelasticity stability in this work is consistent with that recently reported for Ti–Ni based SMAs^50^.

### Isothermal superelasticity stability: at varying and constant applied stress

In view of the significance of transformation stress, transformation reversibility and superelasticity stability, we chose the composition (x = 13.6) with a low transformation stress and narrow transformation hysteresis to investigate its isothermal cyclic superelasticity behaviour under varying and constant applied stress.

**Figure 5:**
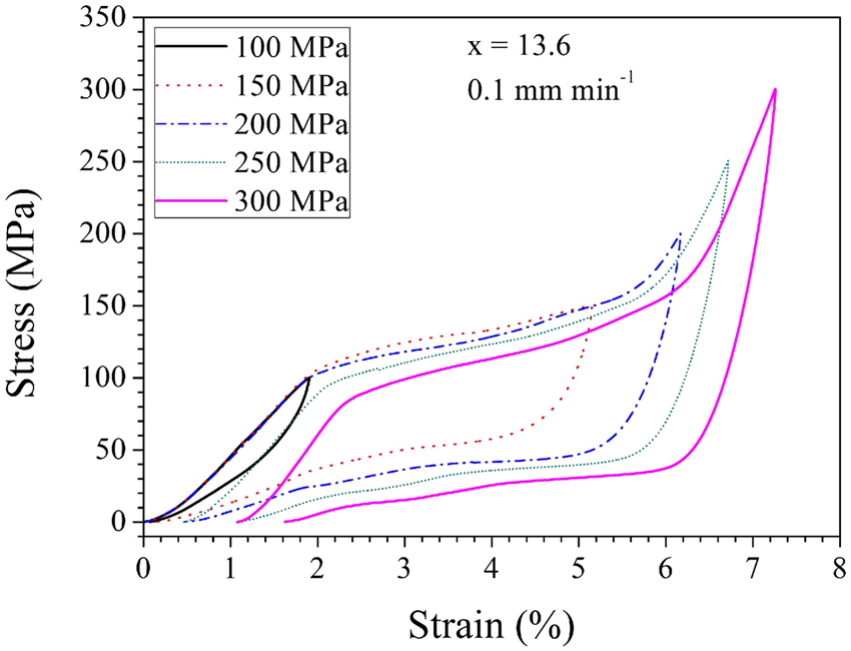
Isothermal stress-strain response upon cycling of Ni_45_Mn_50−x_In_x_Co_5_ (x = 13.6) samples at a strain rate of 0.0003 s^−1^ (0.1 mm min^−1^).

Here, we initially conducted cycling stress–strain experiments with increasing applied stress. All stress–strain tests were performed on a new untrained sample, which remained un-fractured even after a maximum compressive stress of 300 MPa was applied. Figure 5 shows the loading–unloading stress–strain curves for maximum applied stresses between 100 and 300 MPa with a stress interval of 50 MPa. Under an applied stress of 100 MPa, the sample exhibits only the elastic deformation of austenite and the corresponding hysteresis loop is small (0.25 MJ m^−3^). With a higher applied stress of 150 MPa, stress-induced martensite transformation occurs over the plateau stress region between 114 and 150 MPa, leading to a large transformation strain of 3.0%. Upon unloading, the stress-induced martensite transforms back to austenite and the resultant *E*_loss_ reaches 2.8 MJ m^−3^. In the case of an applied stress of 200 MPa, stress-driven martensite transformation begins at 107 MPa and completes at approximately 160 MPa (3.8% transformation strain), followed by elastic deformation of the martensitic phase as an upward trend of the stress-strain curve. However, the release of the applied stress results in a residual strain of 0.5% and a large *E*_loss_ of 4.2 MJ m^−3^. When the applied stress is increased further (250 and 300 MPa), a lower transformation stress plateau but comparable austenite-martensite (A–M) transformation strain (3.9% and 4.0%) is observed upon loading. Upon unloading, nearly identical residual strains (0.6% and 0.6%) and larger hysteresis areas (4.7 MJ m^−3^ and 5.1 MJ m^−3^) are observed for the final two cycles.

The aformentioned stress–strain responses for different applied stresses show that transformation strain reversibility and hysteresis energy loss depend on the magnitude of applied stress and the deformation history. To obtain a large elastocaloric effect, complete stress-induced transformation is desirable. Here, an applied stress of approximately 200 MPa is needed to approach full transformation for the Ni_45_Mn_50−x_In_x_Co_5_ (x = 13.6) sample at 293 K. However, the side effects of applying this stress lie in transformation irreversibility and increased hysteresis energy loss. Therefore, a balance between transformation strain, transformation reversibility and transformation hysteresis is vital to achieving a large and reversible elastocaloric effect. Here a moderate applied stress of 150 MPa is reasonable for x = 13.6.

To achieve a stable elastocaloric temperature change, control of the transformation hysteresis and superelasticity stability during mechanical cycling is important. As demonstrated in Fig. 1, tuning the composition is effective at decreasing stress hysteresis and enhancing the superelasticity stability at various strain rates. However, “mechanical training” may be a feasible way to attain stabilized superelastic behaviour^14^. Here, we investigate the evolution of superelasticity behavior at a constant applied stress under isothermal conditions at 0.00015–0.0015 s^−1^ (0.05–0.5 mm min^−1^). To ensure reversible transformation, an applied stress of ~110 MPa was selected.

**Figure 6:**
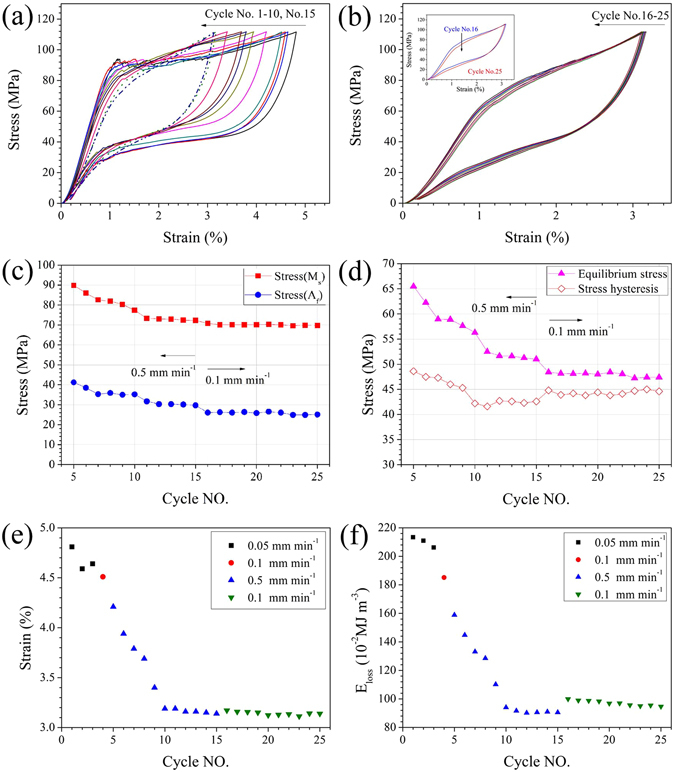
Isothermal stress–strain responses (cycles No.1–15 for (**a**), cycle Nos.16–25 for (**b**)), critical transformation stress (**c**), equilibrium stress and stress hysteresis (**d**), total strain (**e**) and hysteresis energy loss (**f**) upon cycling loading and unloading of Ni_45_Mn_50−x_In_x_Co_5_ (x = 13.6) samples at low strain rates (0.05–0.5 mm min^−1^).

Figure 6 shows the evolutions of typical isothermal stress–strain responses and corresponding characteristic parameters (including transformation stress, transformation strain, transformation hysteresis, and hysteresis loss energy) as a function of the number of cycles. Specifically, several features during cyclic loading–unloading of a virgin sample (x = 13.6) are as follows: (1) The superelastic behaviour tends to stabilize after 15 cycles, and the superelastic response after further 10 cycles with a lower loading/unloading rate (0.1 mm min^−1^) is quite identical to that at the 15^th^ cycle (Fig. 6(a) and (b)). (2) The critical transformation stress *σ*_Ms_ during loading (forward transformation) and *σ*_Af_ during unloading (reverse transformation) decrease with increasing number of cycles (Fig. 6(c)). (3) The slope of the transformation stress plateaus steepen after 6 cycles and stablize at the 15^th^ cycle (Fig. 6(a)). (4) Both the equivalent stress (defined as the average of *σ*_Ms_ and *σ*_Af_) and the stress hysteresis show a tendency to saturate after 15 cycles. However, further cycling at 0.1 mm min^−1^ leads to a decrease in the equivalent stress and a slight increase in the stress hysteresis (Fig. 6(d)). Such a phenomenon is attributed to the more obvious decrease in magnitude of *σ*_Af_ than of *σ*_Ms_. (5) Transformation strain decreases with increasing number of cycles and stablizes after 10 cycles (Fig. 6(e)). (6) Interestingly, the hysteresis energy loss *E*_loss_ exhibits a dramatic decrease from the initial 2.1 MJ m^−3^ (cycle 1) to 0.9 MJ m^−3^ (cycle 10), as shown in (Fig. 6(f)). That is, mechanical training is effective to achieve a stable superelastic response and reduced hysteresis energy loss. The material coefficient of performance (COP_mater_) can be defined by dividing the latent heat *L* by the input mechanical work (ΔW)^11^, whereas the former can be estimated from the adiabatic temperature change (*L* = Δ*T* C_p_ ρ) and the latter can be calculated by integrating the area enclosed by the stress–strain curve (ΔW = *E*_loss_). With increasing numer of cycles, the transformation strain (related to Δ*T* and the latent heat) and hysteresis loss (input mechanical work) decrease and tend to become saturated at the 10^th^ cycle. As a result, mechanical training may provide an effective way to achieve a reproducible elastocaloric response and, finally, a stable COP_mater_.

In summary, the superelastic stability and elastocaloric responses in Ni_45_Mn_50−x_In_x_Co_5_ (x = 13.6, 13.8, 14.0) polycrystalline alloys under a series of strain rates were investigated. The isothermal stress–strain responses show that the stress hysteresis exhibits high compositional sensitivity. The *σ*_Hys_ value for x = 13.6 at 0.0003 s^−1^ is 49 MPa, which is only approximately one-third of that (*σ*_Hys_ = 138 MPa) for x = 13.8. The temperature–time profiles under quasi-adiabatic conditions suggest that the symmetry of the temperature changes upon loading and unloading is strain-rate dependent. Meanwhile, by comparing the evolution of superelastic behaviour under different strain rates among the three compositions, we clearly observed that stress hysteresis affects the superelastic stability. Both the CTS and transformation stress slope are nearly unvaried for the low-hysteresis x = 13.6. Instead, a dramatic decrease in the CTS and an increase in the transformation stress slope occur for the wide-hysteresis x = 13.8. To achieve a sustainable elastocaloric effect, great priority should be given to stable superelastic responses (resulting from narrow stress hysteresis), rather than to large but unsustainable initial elastocaloric temperature changes (originated from wide stress hysteresis). We concudcted isothermal stress–strain cycles for the x = 13.6 polycrystalline sample by increasing the maximum applied stress (100–300 MPa). The obtained transformation parameters, including the transformation strain, residual strain and hysteresis energy loss, depend on the magnitude of the applied stress. A balance among transformation strain, reversibility and hysteresis energy losses is vital to achieving a large and reversible elastocaloric effect.

## Methods

### Sample preparation and magnetic measurements

Polycrystalline ingots with a nominal composition of Ni_45_Mn_50−x_In_x_Co_5_ (x = 13.6, 13.8, 14.0) (at.%) were prepared by arc melting under an argon atmosphere. The ingots were cut into small pieces with approximate dimensions of 3 × 3 × 5.5 mm^3^; the cut specimens were subsequently homogenized at 1173 K for 24 h in a vacuum and then quenched in water. Magnetization versus temperature curves were recorded using a superconducting quantum interference device (SQUID, Quantum Design).

### Measurements of the superelastic response and elastocaloric effect

Compressive tests of the Ni_45_Mn_50−x_In_x_Co_5_ (x = 13.6, 13.8) polycrystalline sample were conducted at ~298 K using a universal testing machine (UTM5105, SUNS). For Ni_45_Mn_36_In_14_Co_5_, a lower testing temperature (T = 287 K) was used to reduce the critical transformation stress (less than the yield strength), enabling adiabatic temperature change tests to be performed. A low strain rate of ~3.0 × 10^−4^ s^−1^ (0.1 mm min^−1^) was chosen to ensure isothermal conditions. The strain was measured using an extensometer. For elastocaloric effect measurement, strain rates ranging from 0.003 to 0.03 s^−1^ (with loading/unloading rates between 1 and 10 mm min^−1^) were selected to approach the adiabatic condition. Each sample was loaded from 0 MPa to the target stress (135, 225, 340 MPa for x = 13.6, 13.8, 14.0, respectively) and subsequently unloaded from the target stress to 0 MPa after the stress was maintianed for 50 s. The temperature changes of the specimens upon loading and unloading were monitored by an adhesive K-type thermocouple pasted onto the specimen surface. The temperature data were recorded at a time interval of 1 s (one point per second).
